# Interleukin-37 exacerbates experimental colitis in an intestinal microbiome-dependent fashion

**DOI:** 10.7150/thno.69616

**Published:** 2022-07-04

**Authors:** Junxiao Cong, Dandan Wu, Hanying Dai, Yanmei Ma, Chenghui Liao, Lingyun Li, Liang Ye, Zhong Huang

**Affiliations:** 1 Department of Immunology, Biological Therapy Institute of Shenzhen University, Guangdong Provincial Key Laboratory of Regional Immunity and Diseases, International Cancer Center, Shenzhen University Health Science Center, Shenzhen, China.; 2 Shenzhen Futian Hospital for Rheumatic Diseases, Shenzhen, China.

**Keywords:** Intestinal microbiota, Inflammatory bowel disease, IL-37, cytokines, immunoregulation

## Abstract

**Background:** Inflammatory bowel disease (IBD) involves complicated crosstalk between host immunity and the gut microbiome, whereas the mechanics of how they govern intestinal inflammation remain poorly understood. In this study, we investigated the contribution of environmental factors to shaping gut microbiota composition in colitis mice that were transgenic for human IL-37, a natural anti-inflammatory cytokine possessing pathogenic and protective functions related to microbiota alterations.

**Methods:** Mice transgenic expressing human IL-37 (IL-37tg) were housed under conventional and specific pathogen-free (SPF) conditions to develop a mouse model of dextran sulfate sodium (DSS)-induced colitis. 16S ribosomal RNA sequencing was used for analyzing fecal microbial communities. The efficacy of microbiota in the development of colitis in IL-37tg mice was investigated after antibiotic treatment and fecal microbiota transplantation (FMT). The mechanism by which IL-37 worsened colitis was studied by evaluating intestinal epithelial barrier function, immune cell infiltration, the expression of diverse cytokines and chemokines, as well as activated signaling pathways.

**Results:** We found that IL-37 overexpression aggravated DSS-induced colitis in conventional mice but protected against colitis in SPF mice. These conflicting results from IL-37tg colitis mice are ascribed to a dysbiosis of the gut microbiota in which detrimental bacteria increased in IL-37tg conventional mice. We further identified that the outcome of IL-37-caused colon inflammation is strongly related to intestinal epithelial barrier impairment caused by pathogenic bacteria, neutrophils, and NK cells recruitment in colon lamina propria and mesenteric lymph node to enhance inflammatory responses in IL-37tg conventional mice.

**Conclusions:** The immunoregulatory properties of IL-37 are detrimental in the face of dysbiosis of the intestinal microbiota, which contributes to exacerbated IBD occurrences that are uncontrollable by the immune system, suggesting that depleting gut pathogenic bacteria or maintaining intestinal microbial and immune homeostasis could be a promising therapeutic strategy for IBD.

## Introduction

Inflammatory bowel disease (IBD) is a multifactorial inflammatory disorder that includes Crohn's disease (CD) and ulcerative colitis (UC), which are characterized by chronic relapsing gut inflammation 1. Although the etiopathogenesis of IBD is not entirely understood, increasing evidence suggests that aberrant immune responses caused by gut microbiome dysbiosis are central underlying themes in the disease 2. The intestinal epithelial barrier is the first physical defensive line, contributing to the maintenance of the commensal microbiota-host immune compartment interface by an intact tight junction 3. In contrast, the impaired intestinal epithelial barrier results in intestinal microbiota dysbiosis accompanied by increased luminal microbial communities that favor pathogenic bacteria over beneficial bacteria, which aggravates the host inflammatory responses to the initiation and development of colitis 4-6. Hence, clarifying the potential crosstalk mechanism between host immunity and microbiome dysbiosis in response to intestinal barrier impairment is urgently required for IBD therapy.

Environmental factors can shape gastrointestinal microbial architecture, impacting susceptibility to intestinal inflammation 7. For instance, mice housed in conventional circumstances are more sensitive to colitis than specific pathogen-free (SPF) animals, implying that distinct microbial communities or members present exclusively in conventionally housed mice affect IBD development 8. Although SPF housing settings have eliminated undesirable possible pathogen impacts that typically occur in conventionally housed mice, their microbiota composition and structure continue to demonstrate a highly variable phenotype 9. In turn, the difference in microbiota composition from different housing situations can influence host responses in IBD. To support this hypothesis, altering microbial composition through the setting of an experimental environment has seen conflicting results in IBD laboratory animals. Mice with interleukin-2 (IL-2) deficiency develop colitis spontaneously in conventional housing conditions, but this is dramatically attenuated in germ-free environments 10, 11. Different environmental communities result in variable disease susceptibility in other mouse IBD models, such as the IL-10-deficient models of acute dextran sulfate sodium (DSS) colitis 12, 13, and the Nlrp6 inflammasome-knockout colitis models 14. As a result, exposure to specific communities has been considered as a critical factor in investigating the interaction between host and microbiota in colitis.

Inflammation triggered by members of the interleukin (IL)-1 family is crucial to the etiology of IBD. The recently discovered IL-1 family member IL-37 has been demonstrated as a negative regulator of innate and adaptive immunity 15, 16. IL-37 is barely expressed in normal tissues and cell lines but strongly increased upon inflammatory stimuli 15, 16. IL-37b is the most potent and well-studied of the five distinct IL-37 splice variants 16. It has been reported that IL-37b possesses a dual-anti-inflammatory mechanism property. Extracellular IL-37 can exert an immunosuppressive role through binding to heterodimeric receptors consisting of IL-18 receptor α (IL-18Rα) and IL-1 receptor 8 (IL-1R8) 17. IL-37 can also translocate to the nucleus depending on caspase-1 cleavage and exert anti-inflammatory action by binding to Smad3 18, 19. Since the mouse lacks a homolog gene for human IL-37, other groups and we developed knock-in mice expressing human IL-37 (IL-37tg) as a tool for investigating the biological function of IL-37* in vivo* 15, 20, 21.

Notably, IL-37 has recently been identified to have dual effectiveness in host protection against infection. In a murine model of infection with *Mycobacterium tuberculosis* and *Aspergillus fumigatus*, overexpression of IL-37 enhanced host resistance infection by suppressing detrimental inflammation 22, 23. In contrast to these findings, IL-37 abrogates the host defense and promotes *Candida albicans and Streptococcus pneumoniae* infection 24-26. These contradictory results proposed that a distinct microbiome might determine the beneficial and detrimental roles of IL-37 in disease progression and outcome by controlling appropriate host immunity. Alternatively, IL-37 may orchestrate particular host immune responses in response to different microbial infections. Although IL-37 can protect against DSS-induced colitis under SPF circumstances 20, 21, whether environmental conditions and specific microbial communities influence the immunoregulatory function of IL-37 in IBD remains unknown.

Here, we investigate the contribution of IL-37 to inflammatory colitis under different housing settings, including conventional housing conditions and SPF conditions. We demonstrate that the transgenic expression of human IL-37 promotes DSS-induced colitis under conventional housing conditions but not under SPF conditions. The detrimental role of IL-37 in conventionally housed colitis mice depends on their intestinal microbiota composition, which is characterized by intestinal epithelial barrier impairment, the infiltration of neutrophils and NK cells, and the upregulation of proinflammatory cytokines. Strikingly, depletion of gut microbiota can substantially improve DSS-induced colitis severity in IL-37tg conventionally housed mice. Our findings indicate that the biological activity of IL-37 is dependent on microbiota communities and that targeting IL-37 or balancing the intestinal microbiota may provide novel therapeutic strategies for modulating IBD.

## Results

### Increased severity of DSS-induced colitis in IL-37tg conventionally housed mice

Previous studies have reported that overexpression of human IL-37 can protect mice against colitis when the animals are fed in specific pathogen-free (SPF) circumstances 20, 21. Of note, in contrast to conventional housing conditions that frequently contain pathogenic bacteria, SPF conditions may influence host immune responses to result in delayed or limited disease development, suggesting that specific microbiomes or distinct communities found only in conventionally housed mice affect disease onset 8. Concurrently, increasing contradictory results from inflammatory bowel disease (IBD) have been linked to the difference in the experimental environment setup 10, 11, 14, 27. To investigate whether transgenic expressing human IL-37 (IL-37tg) mice from various housing environments differ in their susceptibility to intestinal inflammation, we induced experimental colitis with dextran sulfate sodium (DSS) in IL-37tg and wild-type (WT) mice obtained from conventional housing conditions and SPF conditions.

To assess the physiological function of IL-37, we first tested IL-37 expression in colonic epithelial cells following DSS induction. qRT-PCR analysis revealed a significant increase in IL-37 mRNA expression on day 5 and 7 in conventionally housed IL-37tg mice after DSS treatment (Figure S1A). On day 5, we then performed a western blot and confirmed the upregulation of IL-37 protein levels in colonic epithelial cells from these animals (Figure S1B). However, IL-37 expression in intestinal epithelial cells was no different between SPF and conventional housing animals (Figure S1C), implying that the IL-37 production was dependent on inflammatory responses but not the intestinal microbiome.

**Figure 1:**
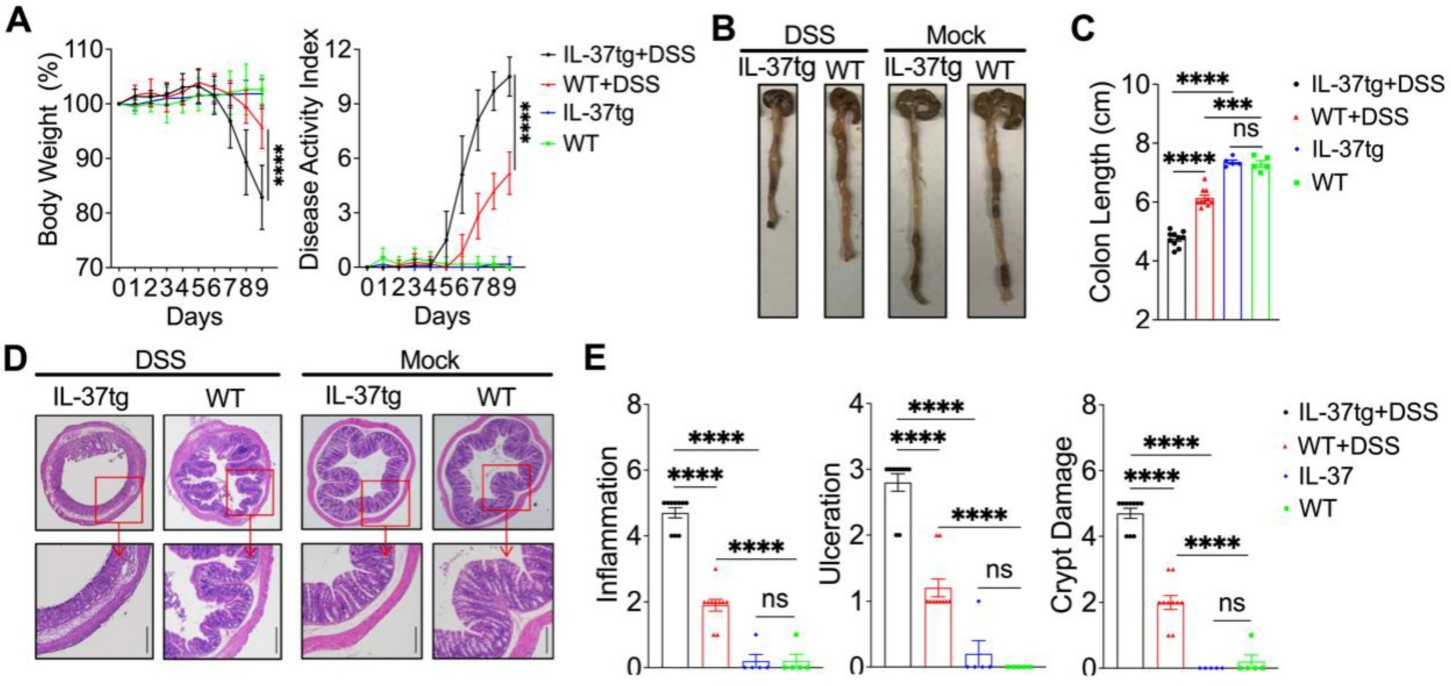
** Overexpressing human IL-37 exacerbates the development of DSS-induced colitis in conventionally bred mice.** WT and IL-37tg conventionally housed mice were given drinking water in the presence (n = 10 in each group) or absence (n = 5 in each group) of 1.5% DSS for eight days, followed by a 24-h recovery period. **(A)** The changes in body weight (left panel) and disease activity index changes (right panel) were monitored daily. **(B)** Macroscopic images of representative mouse colons harvested on day 9. **(C)** The mouse colonic length was assessed on day 9. **(D)** Photomicrographs of an H&E-stained paraffin section of a representative colon from various genotype mice. The lower panels were enlarged from the upper panels. Original magnification × 40 (upper panel) and × 100 (lower panel). **(E)** Histological scores were calculated based on the extent of inflammation, ulceration, and crypt damage for all animals. The data in** (A)** are presented as mean ± SD, while the data in **(C)** and** (E)** are shown as mean ± SEM. *** p < 0.001, **** p < 0.0001, ns = no significant difference by two-way ANOVA with Sidak's multiple comparisons test.

To determine whether IL-37 influences the development of colitis in conventionally housed mice, we induced acute colitis by treating conventionally housed IL-37tg mice and WT littermates with 1.5% DSS for eight days and monitoring them for an additional 24 h. Surprisingly, under conventional housing conditions, IL-37tg colitis mice had a dramatic decrease in body weight and a higher disease activity index, including profuse bloody diarrhea and rectal bleeding between day 7 and 9 (Figure 1A), which coincided with increased colon shortening compared to conventionally housed WT colitis mice on day 9 (Figures 1B and 1C). Correspondingly, there was greater intestinal inflammation and intestinal epithelial damage in conventionally housed IL-37tg mice (Figures 1D and 1E). In steady-state, conventionally housed IL-37tg mice showed stable body weight and healthy colon characteristics without inflammation, identical to WT animals (Figure 1). In contrast, SPF IL-37tg mice demonstrated dramatically diminished inflammation after DSS induction, including effective improvements in body weight, disease activity index, and colonic inflammatory cytokines *Il-1β* and *Il-6* mRNA expression compared to SPF WT mice (Figure S2A-C), consistent with previous reports showing the protective role of IL-37 in colitis mice under SPF conditions. These unexpected findings indicated that IL-37 was detrimental in the DSS colitis model under conventional housing environments.

### IL-37 dysregulates intestinal epithelial barrier of DSS-induced colitis in conventionally housed mice

**Figure 2:**
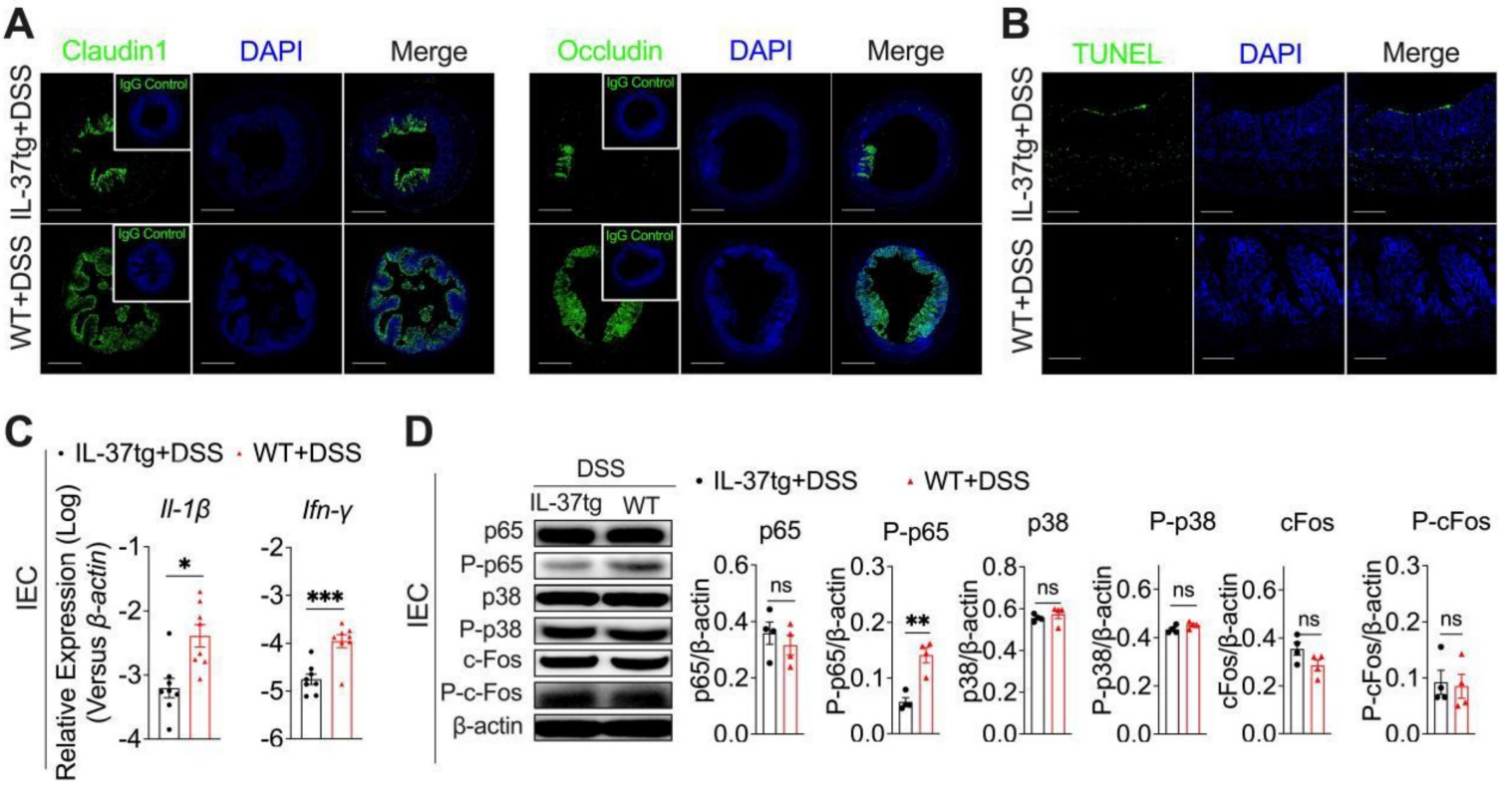
** In DSS-induced conventionally housed mice, IL-37 impairs epithelial tight functions integrity, promotes epithelial cell apoptosis, and suppresses NF-κB-mediated antibacterial gene expression in the colonic epithelium.** Conventionally housed WT and IL-37tg mice were administered 1.5% DSS for eight days. Colon tissues and colonic epithelial cells were extracted on day 9. **(A)** There were representative images of immunohistochemical staining in colonic tissues with antibodies against Claudin1 and Occludin at day eight (Scale bars = 400 μm). Green: positive staining of Claudin1 (left panel), Occludin (right panel), and IgG control (upper right corner); Blue: positive staining of DAPI. IgG control is **(B)** Terminal deoxynucleotidyl transferase dUTP nick-end labeling (TUNEL) staining assay of the colonic epithelium (Scale bars = 100 μm). **(C)** qRT-PCR analysis of *Il-1β* and *Ifn-γ* mRNA expression in colonic intestinal epithelial cells (n = 8 each group). **(D)** Western blot analysis (left panels) and quantification (right panels) of p65, P-p65, p38, P-p38, c-Fos, and P-c-Fos levels in intestinal epithelial cells from conventionally housed WT and IL-37tg mice normalized to β-actin (n = 4 each group). Data are expressed as mean ± SEM. ** p < 0.01; *** p < 0.001, ns = no significant difference by unpaired two-tailed Student's t-test with Welch's correction.

Intestinal epithelial cells (IEC) can prevent the invasion of enteric microbes by forming an integrated physical barrier and maintaining intestinal homeostasis, therefore limiting the development of colitis. Disruption of intestinal epithelial homeostasis due to environmental or other factors can lead to uncontrollable gut microbiota or an aberrant immune response to initiate intestinal inflammation 3. We thus sought to investigate whether IL-37 accelerated DSS-inflamed colitis results from intestinal epithelium barrier disorders and functional disruption. On day nine, the immunofluorescence histochemical analysis of the tight junctional proteins showed that IL-37tg conventional mice had profoundly reduced expression of Claudin1 and Occludin in the colon compared to WT control mice after DSS administration (Figure 2A). Epithelial apoptosis also impairs epithelial barrier integrity 28. We further compared the terminal deoxynucleotidyl transferase dUTP nick-end labeling (TUNEL)-positive apoptosis cells in colon sections of conventionally housed WT and IL-37tg colitis mice by day nine and found increased numbers of TUNEL-positive epithelial cells in IL-37tg colitis mice (Figure 2B), suggesting that IL-37 impairs epithelial tight junction integrity in conventionally housed colitis mice.

We next evaluated the differential gene expression involved in regulating the intestinal epithelial barrier and biological function of conventionally housed IL-37tg and WT mice exposed to DSS. We discovered that colonic epithelial cells isolated from conventionally housed IL-37tg mice after DSS administration had notably lower *interleukin (IL)-1β* and *Interferon (IFN)-γ* expression compared to WT controls (Figure 2C), but other cytokines (*Il-1α, Il-6, Il-10, Il-12, Il-17, Il-18, Gm-csf, Ifn-α, Ifn-β, Ifn-λ2/3, and Tslp*), chemokines (*Ccl2, Ccl3, Ccl4, Ccl5, Ccl20, Cxcl11, and Cx3cl1*), and antibacterial gene (*Camp, Lyz-1, and Lyz-2*) expression in these two genotype mice were largely unaffected (Figure S3A-C). To better understand how IL-37 regulates these two cytokine responses in IEC during DSS induction, we next examined the activities of NF-κB and MAPK, the crucial effector molecules for driving IL-1β and IFN-γ signaling 17, 29. The western blot analysis of phosphorylated-p65 (P-p65), the primary signaling molecule required for NF-κB activation, revealed about threefold fewer activation of P-p65 in the IEC of conventionally housed IL-37tg colitis mice than in their conventionally housed WT counterparts (Figures 2D). Furthermore, we observed no difference between conventionally housed IL-37tg colitis mice and WT colitis mice in their phosphorylation and mRNA expression of p38 and c-Fos, the key mediators for MAPK pathways (Figures 2D, Figure S3D). In conventionally housed IL-37tg mice with DSS induction, IL-37 receptors *Il-18r1* and *Il1rapl1* mRNA levels and additional downstream signaling pathway genes (*Jun, Myd88, Sigirr, Smad3, Stat1, and Stat3*) were expressed similarly as in control WT colitis mice (Figure S3D). Under static conditions, however, IL-37 neither affects the expression of proinflammatory cytokines (*Il-1β, Il-6,* and *Ifn-γ)* in IEC nor modulates p65 and P-p65 production in the intestinal epithelium (Figure S4A-B). IL-1β and IFN-γ have been reported to promote IEC proliferation and antibacterial defense 30, 31. Therefore, these experiments indicated that the deterioration of the intestinal epithelium barrier induced by IL-37 in DSS-induced colitis under conventional housing circumstances involves the downregulation of IL-1β and IFN-γ expression, which is dependent on NF-κB signaling.

### IL-37 enhanced colonic inflammation by recruiting neutrophils and NK cells and inducing proinflammatory cytokine expression

**Figure 3:**
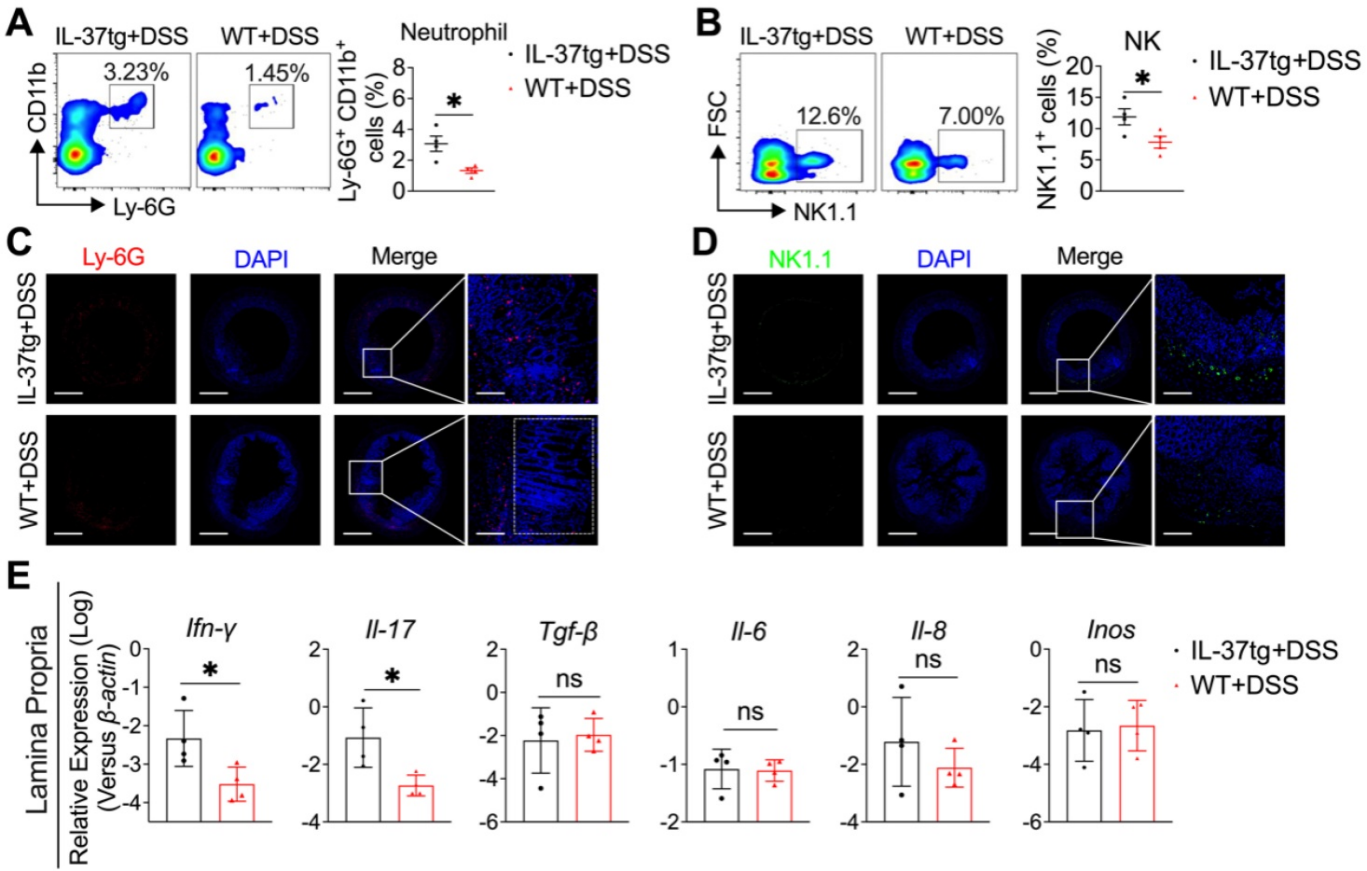
** Conventionally housed IL-37tg mice showed increased inflammation in the colon characterized by increasing neutrophils and NK cell infiltration and proinflammatory cytokine expression.** Colon and lamina propria were isolated at day nine from 1.5% DSS-induced WT and IL-37tg colitis mice kept under conventional housing conditions. **(A-B)** On day nine, flow cytometric analysis of the percentage of CD11b^+^Ly6G^+^ neutrophils **(A)** and NK1.1^+^ NK cells **(B)** in mesenteric lymph nodes (MLN). Each symbol represents an individual animal. **(C-D)** Representative immunofluorescence photomicrographs of the colon stained with Ly-6G for neutrophils **(C**, red**)**, NK1.1 for NK cells **(D**, red**)**, and DAPI **(C** and **D**, blue**)**. Merged pictures are shown at low magnification (left panels, scale bars = 400 μm), and boxed areas are presented at higher magnification (right panels, scale bars = 150 μm). **(E)** The mRNA expression of *Ifn-γ, Il-17, Il-22, Tgf-β, Il-6, Il-8*, and* Inos* in colon lamina propria was determined by qRT-PCR. The results are representative of two independent experiments and are shown as mean ± SEM. * p < 0.05; ** p < 0.01, ns = no significant difference by unpaired two-tailed Student's t-test with Welch's correction.

To further understand the mechanism by which IL-37 promotes inflammation in the colon, we first evaluated the immunological events by flow cytometry in the mesenteric lymph node (MLN) in WT and IL-37tg mice kept under conventional conditions without DSS induction. We found that IL-37 did not affect immune cell composition in MLN at a steady status (Figure S4C). Interestingly, following DSS induction, we noticed that the MLN of IL-37tg conventional mice contained roughly 2-fold more CD11b^+^Ly6-G^+^ neutrophils (Figure 3A) and 1.5-fold more NK1.1^+^ NK cells (Figure 3B) than WT mice. However, the frequencies of CD4^+^ T cells, CD8^+^ T cells, Ly6c^+^ monocytes, CD19^+^ B cells, CD11c^+^MHC-II^+^ DCs, and CD11b^+^F4/80^+^ macrophages in MLN are comparable between IL-37tg conventional mice and WT littermate controls (Figure S5). Consistent with the observation in MLN, the accumulation of neutrophils (Figure 3C) and NK cells (Figure 3D) was considerably increased in the colon lamina propria (CLP) of conventionally housed IL-37tg colitis mice compared with that of conventionally housed WT colitis mice. To investigate possible inflammatory changes in the colon of DSS-treated IL-37tg conventionally housed mice, we assessed gene expression in the CLP by quantitative real-time PCR. We found higher expression of genes encoding proinflammatory molecules *Ifn-γ* and *Il-17* in the CLP of conventionally housed IL-37tg mice subjected to DSS than in their WT counterparts (Figure 3E), whereas the expression of *Tgf-β*, *Il-6*, *Il-8*, and *Inos* were unaffected (Figure 3E). These findings demonstrated that the characteristics of colonic inflammation displayed by IL-37tg mice under conventional housing circumstances are strongly related to neutrophils and NK cells infiltration in MLN and CLP and the expression of proinflammatory cytokines IFN-γ and IL-17.

### IL-37 influences gut microbiota composition in DSS-induced conventional housing mice

**Figure 4:**
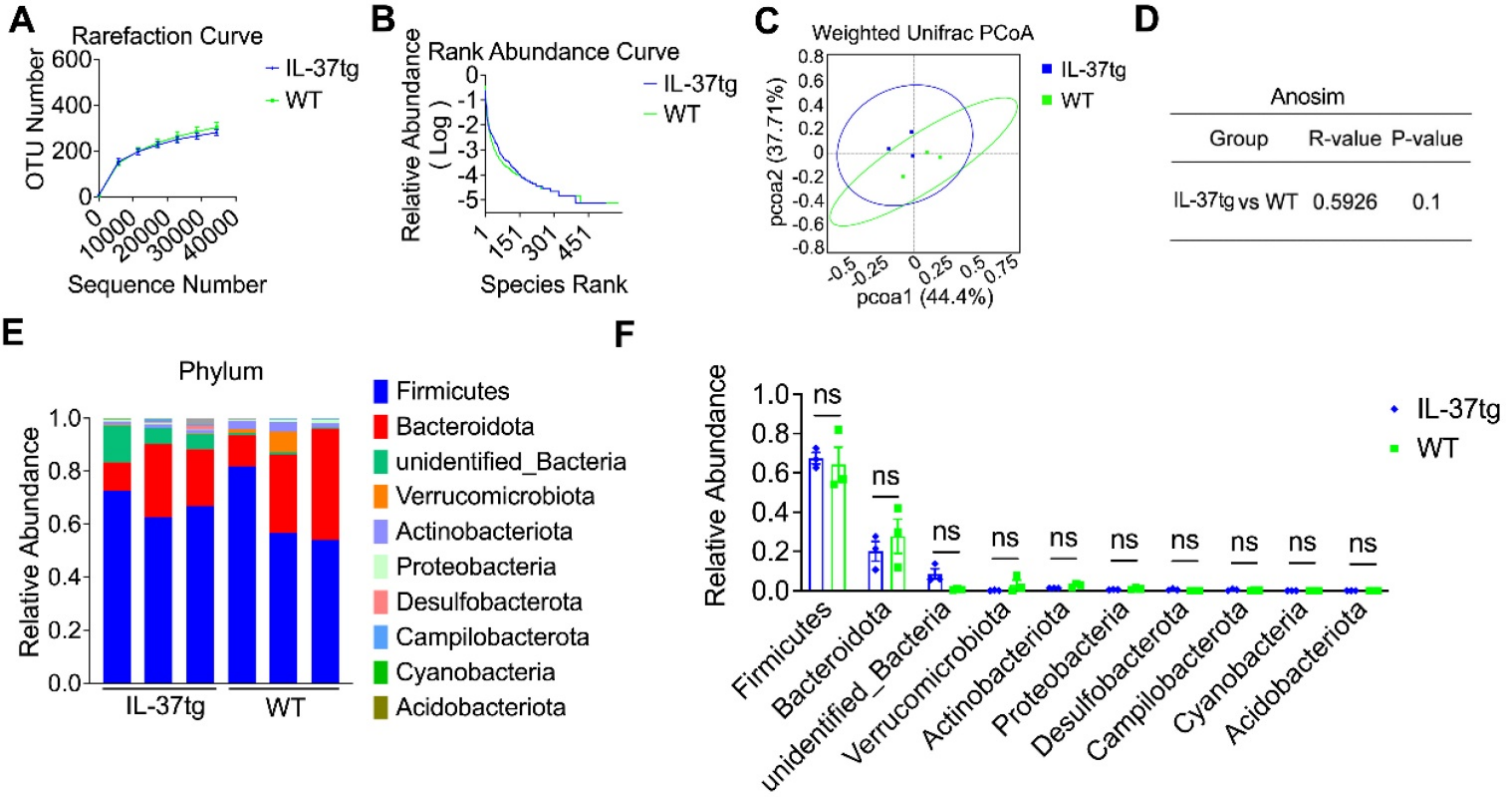
** Comparison of gut microbiota of steady-state WT and IL-37tg conventionally housed mice by 16S ribosomal RNA sequencing. (A-D)** Rarefaction curve based on operational taxonomic units (OTUs) **(A)**, rank abundance curve **(B)**, principal coordinates analysis (PCoA) **(C)**, and anosim analysis **(D)** of feces isolated from naïve WT (n = 3) and IL-37tg mice (n = 3) by 16S rRNA sequencing. P = 0.1 by unpaired two-tailed Students's test. **(E-F)** Compositions of relative abundance of microbiota at phylum level in the fecal samples from naïve WT (n = 3) and IL-37tg conventional mice (n = 3). Individual samples of each group **(E)**; intra-group results **(F)**. Data are shown as mean ± SEM. ns = no significant difference by two-way ANOVA with Sidak's multiple comparisons test.

Accumulating evidence supports that DSS-induced chemical impairment of the IEC barrier can result in gut microbiome dysregulation and intestinal homeostasis disruption 32. We propose that IL-37-aggravated intestinal inflammation under conventional housing conditions might be associated with gut microbiome dysregulation. To first assess the effect of IL-37 in regulating the gut microbiome community at steady-state, fecal samples of conventionally housed naïve IL-37tg mice and their WT counterparts were collected for 16S ribosomal RNA analysis. The rarefaction curve based on operational taxonomic units (OTUs) and rank abundance curve showed that relative species abundance and diversity of gut communities in conventionally housed naïve IL-37tg mice are pretty similar to WT animals (Figure 4A-B). Weighted Unifrac-based principal coordinate analysis (PCoA) and analysis of similarities (Anosim) indicated no apparent separation between the fecal microbiota of conventionally housed IL-37tg mice and conventionally housed WT mice at steady-state (Figure 4C-D). Compared with that observed in the naïve WT conventional mice, the abundance of gut microbiota at phylum levels was not statistically significant in the naïve IL-37tg conventional mice (Figure 4E-F). These results showed that IL-37tg conventional mice harbored a regular gut microbiota community before DSS induction.

**Figure 5:**
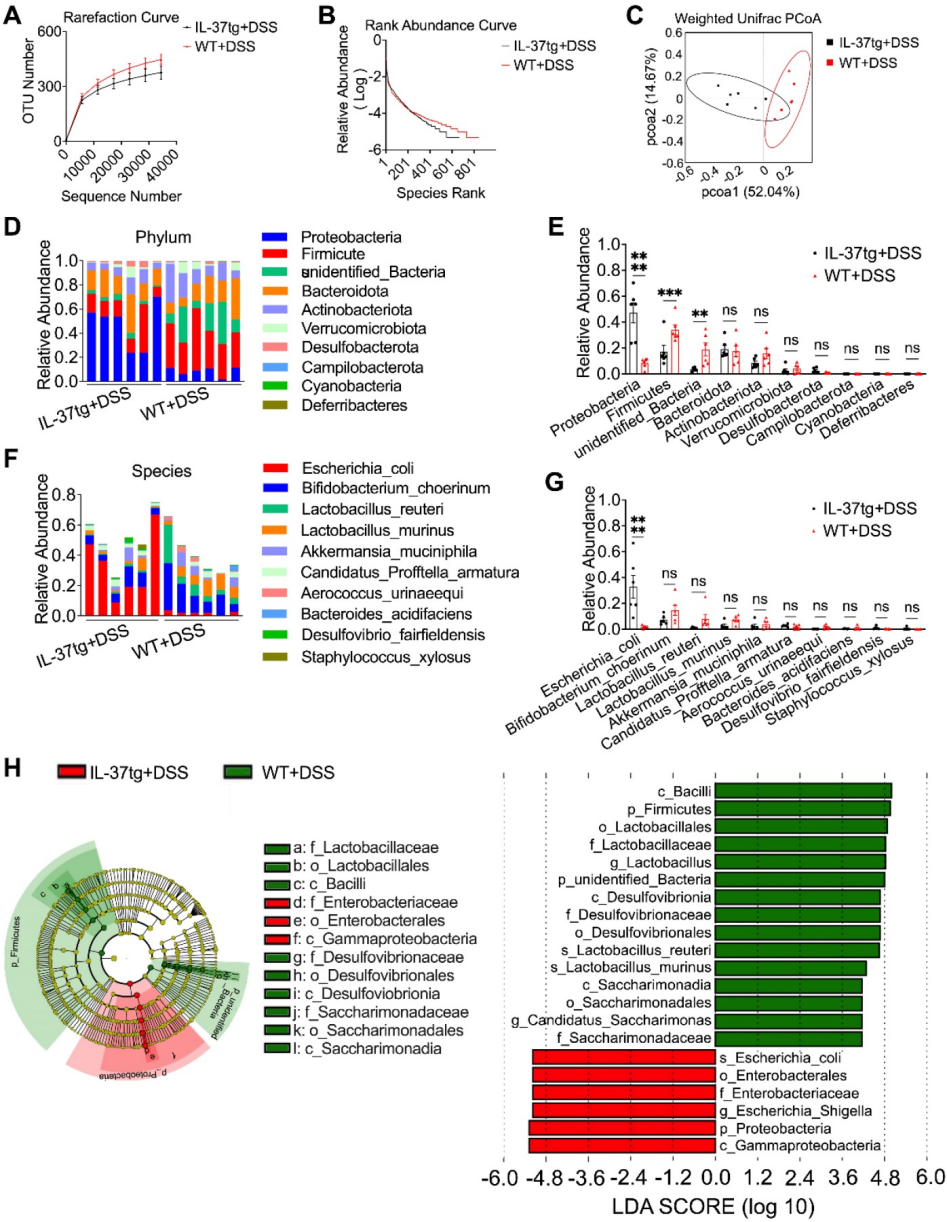
** Transgenic expression of human IL-37 alters the intestinal microbiota signature in DSS-induced colitis mice kept under conventional housing conditions.** Conventionally housed WT and IL-37tg mice were administered with 1.5% DSS dissolved in drinking water for seven days, and feces were collected from day 4 to day 8 for 16S ribosomal RNA gene sequencing. Rarefaction curves based on operational taxonomic units (OTUs) **(A)** and rank abundance distribution curve analysis **(B)** in feces between conventionally housed WT and IL-37tg mice. **(C)** Weighted unifrac principal coordinates analysis (PCoA) plot of the gut microbiota composition from different genotype mice (n = 6 in each group). **(D-E)** Relative abundance of intestinal flora from DSS-treated WT and IL-37tg conventionally housed mice on Phylum level. Individual samples of each group **(D)**; intra-group results **(E)**. **(F-G)** Species-level comparison of intestinal microbiota in conventionally housed WT and IL-37tg mice. **(F)** Individual samples of each group; **(G)** intra-group results. **(H)** Cladogram plotted from LEfSe analysis of showing the taxonomic levels represented by rings with phyla in the outermost ring and genera in the innermost ring **(H**, left panel**)**. Each circle represents a member within that level. Comparison of the relative abundance of fecal bacteria between WT and IL-37tg groups using LEfSe analysis **(H**, right panel**)**. Taxa satisfying an LDA significant threshold are shown, taxa enriched in conventionally housed WT colitis mice (green) and conventionally housed IL-37tg colitis mice enriched in taxa (red). LEfSe, linear discriminant analysis effect size. Data are shown as mean ± SEM. **p < 0.01, *** p < 0.001, **** p < 0.0001, ns = no significant difference by two-way ANOVA with Sidak's multiple comparisons test.

Since the gut microbiota composition of steady-state IL-37tg mice and their WT counterparts was comparable under conventional housing conditions, we next investigated whether IL-37 contributes to the gut microbiota dysbiosis in an IBD mouse model under similar conditions. We performed 16S ribosomal RNA analysis on fecal bacterial DNA isolated from IL-37tg and WT conventionally housed mice following DSS induction. We observed that the fecal of IL-37tg conventional housing mice contained decreased OTUs and had lower microbial richness than WT control mice after induction of colitis (Figure 5A-B). The weighted Unifrac-based PCoA revealed that IL-37tg conventionally housed animals with DSS induction had a shift in fecal bacterial composition clustering that was distinct from WT animals (Figure 5C), indicating that IL-37 caused the dysbiosis of gut microbiota in colitis. Further analysis of the gut bacterial composition at the phylum level revealed that IL-37 mainly increased the relative abundance of *Proteobacteria* and significantly decreased the relative abundance of *Firmicutes* and other unidentified bacteria (Figure 5D-E). Furthermore, extended analysis at the species level demonstrated that IL-37 mainly increased the abundance of *Escherichia coli* (*E. coli*), a common pathogenic strain of the gut microbiota dysbiosis in DSS-induced colitis (Figure 5F-G). However, the abundance of the remaining species did not differ substantially between each group. Consistent with the above results, linear discriminant analysis effect size analysis measurements (LEfSe) revealed that *E. coli* from the proteobacteria phylum were enriched in the IL-37tg mice group, whereas *Firmicutes* and some unidentified bacteria were more abundant in the WT group (Figure 5H). However, no significant differences in *E. coli* abundance were observed between the SPF IL-37tg and SPF WT mice after DSS induction (Figure S6A-B). Thus, our data suggest that overexpression of exogenous IL-37 leads to gut microbiota dysbiosis in DSS-induced conventionally housed mice.

### Intestinal microbiota determines the severity of DSS-induced colitis in IL-37tg conventionally housed mice

**Figure 6:**
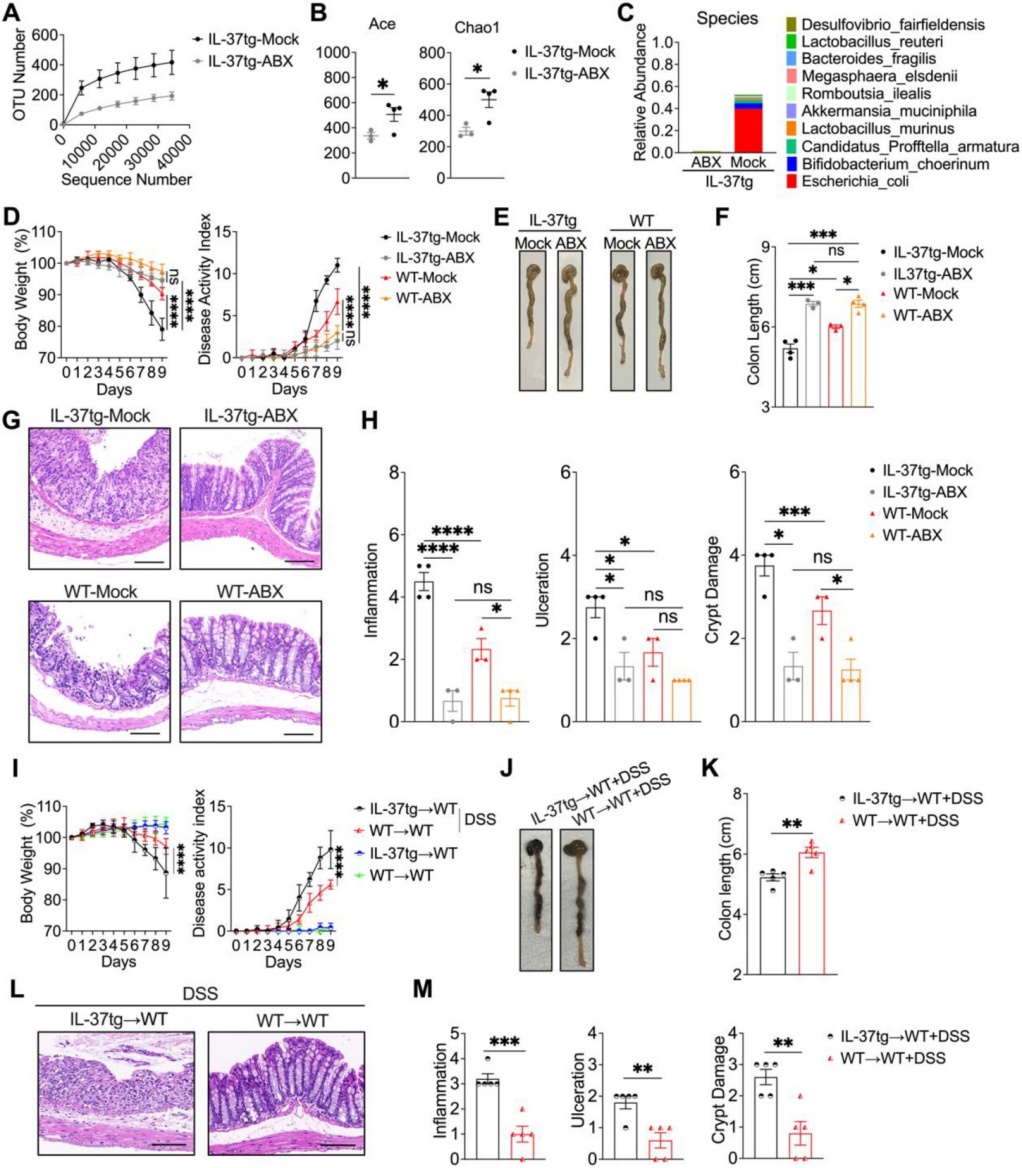
** The increased severity of colitis in conventionally housed IL-37tg mice is intestinal microbe dependent.** For antibiotic treatment experiment, conventionally housed IL-37tg or WT mice were pretreated with (ABX) or without (Mock) 200 μL antibiotic cocktail (ampicillin sodium 2 g/L, metronidazole 2 g/L, neomycin 2 g/L, gentamicin 2 g/L, and vancomycin 1 g/L) for consecutive ten days. Afterwards, all animals were given 1.5% DSS in drinking water for eight days before switching to water without DSS to drink for one day. For FMT experiment, IL-37tg and WT conventionally housed mice (Donors) were given 1.5% DSS in drinking water for 8 days. Stool and colonic contents were collected and processed for transplantation. Conventionally housed recipient WT mice were pretreated with an antibiotic cocktail (ampicillin sodium 2 g/L, metronidazole 2 g/L, neomycin 2 g/L, gentamicin 2 g/L, and vancomycin 1 g/L) once every two days for five times before receiving microbiota samples from donor mice after two days of DSS administration. Analysis of rarefaction curves of operational taxonomic units (OTUs)** (A)**, relative abundance (Abundance-based coverage estimator (ACE) and Chao indices (Chao1) **(B)**, relative abundance at the species level **(C)** of gut microbiota from different groups using 16S rRNA sequencing. **(D, I)** Body weight and disease activity index in mice were monitored from day 0 to day 9. **(E-F)** Representative images of mouse colons **(E, J)** and colonic length **(F, K)** were assessed on day 9. **(G, L)** Representative hematoxylin and eosin (H&E) staining images in the colon (Scale bar = 200 μm). **(H, M)** Histological scores were evaluated based on the extent of inflammation, ulceration, and crypt damage for all animals. Data from **A, D and I** are shown as mean ± SD. **** p < 0.0001, ns = no significant by Sidak's multiple comparisons test. Data in** B**, **F**, **H, K and M** are shown as mean ± SEM. * p < 0.05, ** p < 0.01, *** p < 0.001, **** p < 0.0001, ns = no significant by unpaired two-tailed Student's t-test with Welch's correction.

To determine whether intestinal bacteria contribute to the colitis phenotype of conventionally housed IL-37tg mice, animals were given an antibiotic cocktail for a consecutive ten days prior to DSS induction. 16S ribosomal RNA analysis in gut microbiota showed that antibiotic-treated conventionally housed IL-37tg mice had obviously decreased bacterial richness based on OTUs and alpha-diversity analysis using the Ace index and Chao1 index after colitis induction (Figures 6A-B). We further observed that gut communities at the species level are completely abrogated in conventionally housed IL-37tg colitis mice after antibiotic treatment, especially the most predominant *E. coli* in antibiotic treated conventionally housed IL-37tg mice have not appeared (Figures 6C). As a result, our data revealed that the intestinal microbiota, particularly* E. coli* from the *Proteobacteria*, was effectively depleted in conventionally housed IL-37tg mice by antibiotic treatment.

Next, we evaluated whether depletion of the gut microbiota in conventionally reared IL-37tg mice affected the development of colitis in the mice. In comparison to conventionally housed control mice, antibiotic-treated conventionally housed IL-37tg mice had less weight loss and colitis symptoms, including profuse bloody diarrhea and rectal bleeding, resulting in a lower disease activity index (Figure 6D). These observations also coincided with improved colon length in conventionally housed IL-37tg mice following antibiotic administration (Figures 6E-F). Moreover, administration of antibiotic cocktails remarkably reduced the inflammation and improved IEC structure in conventionally housed IL-37tg mice compared to control animals (Figures 6G-H). These results indicated that antibiotic treatment ameliorates DSS-induced colitis in IL-37tg conventional mice.

To directly demonstrate that the altered gut microbiota in conventionally housed IL-37tg mice was responsible for the susceptibility to colitis, we performed a fecal microbiota transplantation (FMT) experiment in which gut microbiota-depleted WT mice (WT recipients) were reconstituted with the gut microbiota of conventionally housed IL-37tg or WT donor mice (IL-37tg donors or WT donors) with DSS induction. In the absence of DSS induction, WT recipients that received fecal from either IL-37tg or WT colitis donors did not show any difference in body weight or disease activity index (Figures 6I). However, we observed a significant decrease in body weight and a significant increase in disease activity index in WT recipients that received fecal from IL-37tg colitis donors compared with WT recipients that received from WT colitis donors after DSS administration (Figures 6I-K). Similarly, fecal materials from IL-37tg colitis donors caused more severe colitis than fecal material from WT colitis donors as determined by more severe intestinal inflammation and intestinal epithelial damage (Figures 6L-M). Collectively, these results indicate that the increased severity of colitis in conventionally housed IL-37tg mice appears to be intestinal microbe dependent, with preexisting IEC dysfunction being a key contributing factor.

## Discussion

Healthy gut homeostasis is inseparable from the maintenance and regulation of the microbiota, the host immune system, and the intestinal epithelial barrier 2. Microbial dysbiosis has been demonstrated to contribute to inflamed colitis upon intestinal epithelial barrier impairment. Furthermore, intestinal epithelial barrier dysfunction has been identified as a signature in IBD patients. It can cause colon inflammation by allowing more bacteria to translocate to the laminal propria compartment and triggering inappropriate host immune responses when confronted with the commensal microbiota 3-6. However, the relative role and contribution of host—gut microbiota and the underlying mechanism by which the intestinal epithelial barrier orchestrates the intricate interplay between the immune system and the microbiota in the pathogenesis of IBD remains poorly unknown. The present study provides experimental evidence that transgenics expressing human IL-37 aggravate DSS-induced colitis, which is strongly linked to intestinal barrier defects and pathogenic bacteria invasion, both of which might promote immune cell recruitment to exacerbate inflammatory responses. However, it should be highlighted that the colitis induced by IL-37 was only observed in experimental animals maintained in conventional facilities, whereas the protective effects were observed in colitis under SPF experimental settings. This is in line with previous observations that IL-37 derived either from hematopoietic cells, or mesenchymal stromal cells, or T cells, is sufficient to curb colon inflammation in SPF mice 20, 21, 33, 34. Indeed, the protective and pathogenic functions of IL-37 during colitis were consistent with those of other members of the IL-1 family, including IL-1, IL-33, and IL-36 35-38. These contradictory results indicated that IL-37 had both beneficial and adverse effects on intestinal inflammation under specific experimental situations, which could be attributed to changes in the gut microbiome. Alterations in the microbiota community triggered phenotypic disparities in immune system resistance diseases have been identified in experimental animals with the same genotype but maintained in different facilities 10-12, 14, 27. Analysis of the gut microbiota composition by 16S rRNA sequencing demonstrated that under conventionally housed conditions, bacterial phylum levels of *Proteobacteria* were abundant in IL-37tg mice, but there was a remarkable reduction in commensal *Firmicutes*. These findings are consistent with previous observations that IBD patients had an imbalance of *Proteobacteria* and* Firmicutes*, with *Proteobacteria* outnumbering *Firmicutes* 39. We further noted that *E. coli* from the *proteobacteria* phylum was preferentially increased in conventionally housed IL-37tg mice. Of note, *E. coli* has been shown as a pathogenic cause of active colitis, facilitating intestinal inflammation 40. Surprisingly, eliminating microbiota by antibiotics can restore the balance of gut microbiota, particularly entirely depleted *E. coli*, which improves the severity of colon inflammation in conventionally housed IL-37tg mice. In contrast, fecal microbiota transplantation from conventionally housed IL-37tg mice aggravates colon inflammation. Therefore, these results support the hypothesis that intestinal microbiota dysbiosis is responsible for exacerbating colon inflammation in IL-37tg mice under conventional housing conditions.

Environmental variables can strongly influence the microbiome composition, leading to the opposite outcome in animal experiments. Here, our findings confirmed that IL-37 has a dual effect in colitis by shaping intestinal flora composition when animals are housed in conventional or SPF conditions. It is commonly accepted that conventionally housed mice with different microbiome communities are a valuable tool for studying the impact of diverse microbial populations on disease development 7, 41. SPF mice are absent pathogens commonly seen in conventionally housed laboratory mice in general, which may not fully reveal or conceal the causality of the diseases resulting from specific microbiota and alter host immune function 8. Following this notion, conventionally housed TNF^deltaARE^ mice with a deletion in the TNF AU-rich (adenosin-uracil) elements (ARE) can develop colitis but do not exhibit indications of colon inflammation in SPF or germ-free facilities 27. Antibiotic treatment significantly attenuates the severity of colitis in conventionally housed TNF^deltaARE^ animals, accompanied by a shift in bacterial distribution, with an increase in *Firmicutes* and a decrease in *Bacteroidetes* 27, indicating the importance of the gut bacterial community in regulating host immunity against colitis. Similar conflicting findings have been reported utilizing other genetic background animals, such as Nlrp6 knockout mice 14 and IL-10 knockout mice 12, 13. Thus, our data corroborated these concepts, demonstrating that IL-37 promotes the pathology of colitis depending on microbiota dysbiosis, and future studies into the interaction of host and microbiota composition need to consider the environmental changes.

Astonishingly, a failure to identify differences in intestinal microbiome structure and composition between WT and IL-37tg conventional mice prior to DSS induction. Given that DSS is essential for mouse colitis modes by causing mucosal injury and barrier dysfunction, permitting microbial translocation^32^ , we may reasonably anticipate that the disorder of the intestinal epithelial barrier may involve the imbalance of the gut flora in conventionally housed IL-37tg colitis mice. As expected, we found that IL-37 significantly downregulated Claudin1 and Occludin, two components of tight junctions associated with the maintenance of IEC permeability and barrier function 42. Moreover, under this experimental setting, events of IEC apoptosis were observed in IL-37tg colitis mice, indicating that IEC apoptosis is a feature of IL-37-promoted intestinal epithelial barrier dysfunction. A defective tight junction barrier is a key pathogenic factor for colitis involving exposure opportunity to the luminal bacterial environment and altered intestinal microbiota composition, suggesting IEC barrier disruption and apoptosis may contribute to microbiota dysbiosis of conventionally housed IL-37tg mice upon DSS induction. In addition to forming a physical interface as a first-line of defense, IEC has the potential to release several cytokines and chemokines that could impact tight junctions or respond to luminal bacteria 3. IEC can also produce antimicrobial molecules to defend luminal bacterium adherence. Our findings show that IL-37 predominantly restrains IL-1β and IFN-γ expression in IEC, but not other cytokines, host factors, chemokines, and antibacterial components. This is in accordance with previous notions from animal experiments and human samples that IL-37 can inhibit the production of proinflammatory cytokines (IL-1β and IFN-γ) in the IEC 15, 16. Additionally, IL-37 can downregulate intracellular p38 mitogen-activated protein kinase (MAPK) or nuclear factor κB (NF-κB) activation-mediated proinflammatory signaling 16, 17, 21, 43, 44, but our findings show that the inhibitory action of IL-37 in IEC is closely associated with NF-κB activation rather than the MAPK signaling pathway. It is notable that IL-1β and IFN-γ have been shown to exert antimicrobial effector mechanisms at mucosal surfaces 45-48. Recently studies reported that IL-37 has a detrimental influence on host defense against infection by restraining the production of such proinflammatory cytokines 24-26, 49. These results provide insights into the involvement of IL-37 in triggering intestinal microbiota dysbiosis following IEC injury caused by DSS induction, which is pertinent with the suppression of NF-κB mediated IL-1β and IFN-γ antibacterial effectors. Further assessment of the individual or synthetic antibacterial effects of IL-1β and IFN-γ in this experimental model will yield more direct and solid evidence for a better understanding of the action mechanism of IL-37 in disrupting the intestinal epithelial barrier.

Colitis occurrence involves alteration in the migration of immune cells to exacerbate colon inflammation. Here, we found a substantial infiltration of NK and neutrophils but not other immune cells in MLN and CLP from conventionally housed IL-37tg colitis mice, indicating that selective recruitment of these cells may provide an inflammatory amplification loop in the colon. Uncontrolled production of IL-17 and IFN-γ is involved in the pathogenesis of intestinal inflammation^50^, and IFN-γ can lead to increase IL-17 production and neutrophil infiltration^51^. Mechanistically, we observed increased expression of cytokines IL-17 and IFN-γ in colonic homogenates of IL-37tg mice under conventionally housed conditions, which may explain why IL-37tg mice show accelerated colon inflammation. Although the cellular sources of IFN-γ production in the MLN and CLP have not been resolved in this investigation, conventional wisdom holds that IFN-γ is produced by NK cells in response to bacterial infection 52. Correspondingly, we observed an increased harmful bacterial profile in IL-37tg mice during DSS-induced colitis under the conventional facility, implying that NK cells in these animals might not be able to effectively eliminate bacteria and attract neutrophils to reach the inflamed site, resulting in excessive inflammation by releasing IL-17. Since the mechanism of immune cell crosstalk is a great enigma, it will be fascinating to determine how IL-37 modulates the interaction of NK cells and neutrophils in this experiment and whether blocking these inflammatory signals can protect against IL-37tg mouse colitis.

Taken together, our study indicates that the potent anti-inflammatory properties of IL-37 are deleterious during colitis in conventional housing settings. Mechanistically, the adverse effects of IL-37 in this mouse model are attributed to the intestinal barrier destruction causing intestinal microbiome dysbiosis, which leads to an uncontrollable enteritis incident by accumulating NK and neutrophils. Restoring intestinal homeostasis, on the other hand, can accomplish the protective effect of IL-37 in colitis. This finding has implications for understanding the interplay of host immunity and microbiota during human disease and provides a rationale for exploring immunotherapy strategies that take microbiota homeostasis into account. However, this study based on our mouse model experiments remains to be verified in clinical trials.

## Materials and Methods

### Mice

The generation of transgenic expressing human IL-37 (IL-37tg) mice has been previously described. Wild-type (WT) and IL-37tg mice with C57BL/6 genetic backgrounds were bred under standard housing conditions or specific pathogen-free (SPF) conditions at the Shenzhen University animal facility. All animal experiments were approved by the Institutional Animal Care and Use Committee at Shenzhen University. The genotype of the mice was verified by PCR analysis of tail tip digests both before and after sacrifice. Fecal pellets were collected for RNA isolation and 16S ribosomal RNA gene sequence analysis.

### Mouse model of DSS-induced colitis

To induce acute colitis, Female WT and IL-37tg mice (Seven to nine weeks old) raised at conventional housing conditions or SPF conditions were given 1.5% (w/v) dextran sulfate sodium (DSS) (MP Biomedicals, Canada) in distilled water for 8 days, followed by additional 24 h of administration of regular water. Body weight, Diarrhea and bloody stool were monitored daily for disease activity index (DAI) assessment. Mice were sacrificed, and samples were harvested at indicated time point after DSS induction. Colon tissues were collected for further analyses. Fresh fecal samples of conventionally housed WT and IL-37tg colitis mice were frozen in liquid nitrogen after collection, and then stored at -80 °C for following 16S rRNA sequencing.

### Antibiotic treatment in the colitis mouse model

Female conventionally housed IL-37tg and WT mice were orally gavaged with 200 μL of antibiotic cocktails (ABX, Ampicillin sodium 2 g/L, Metronidazole 2 g/L, Neomycin 2 g/L, Gentamicin 2 g/L, Vancomycin 1 g/L) for 10 days. Afterwards, mice were given 1.5% (w/v) DSS (MP Biomedicals, Canada) in regular water for 8 consecutive days, followed by regular water for one day. Mice were sacrificed for analysis on day 9 of related experiments. Fecal pellets of mice with or without antibiotic treatment were collected and stored at -80 °C until 16S rRNA sequencing.

### Fecal Microbiota Transplantation

IL-37tg and WT conventionally housed mice (Donors) were given 1.5% DSS in drinking water for 8 days. Fresh stool and colonic contents were collected from donor mice and immediately transferred into anaerobic tubes filled with inert gas. 1 g Obtained samples were homogenized in 1 mL LYH-BHI medium (Brain-heart infusion broth 37 g/L, Yeast extract 5 g/L, Hemin 5 mg/L, Cellobiose 1 g/L, Maltose 1 g/L, Cysteine 0.5 g/L), and filtered through 70 μm strainers for frozen. WT mice from the recipient set were intragastrically given antibiotic cocktails (ABX, Ampicillin sodium 2 g/L, Metronidazole 2 g/L, Neomycin 2 g/L, Gentamicin 2 g/L, Vancomycin 1 g/L) once every other day for five times. Six days after ABX treatment, drinking water for recipient mice was switched to a 1.5% DSS solution. Recipient mice were daily treated with 200 μL transplantation samples from different donor groups by oral gavage from day 2 of DSS induction. All microbiota samples were thawed within 10 min before transplantation to avoid alteration of microbiota composition.

### Microbiota 16S rRNA gene sequencing

The genomic DNA of stool samples was extracted by conventional cetyltrimethylammonium bromide (CTAB) method. The v4 regions of the bacteria 16S rRNA gene were amplified by PCR using primers. Library was constructed according to TruSeq® DNA PCR-Free Sample Preparation Kit for library construction, and the 16S rDNA amplicons were sequenced on an Illumina NovaSeq6000 platform. Based on 97% nucleotide identity of the amplicon sequences, reads were clustered into operational taxonomical units (OTUs). Sequence data analyses were performed by QIIME and the R packages. Following the annotation of each species, relative abundance analysis, Alpha Diversity, and Beta Diversity were performed.

### The disease activity index (DAI)

DAI was determined by scoring the degree of body weight loss (0 = no loss; 1 = 1%-5% loss; 2 = 5%-10% loss; 3 = 10%-15% loss;4 > 15% loss), diarrhea (0 = normal; 1 = slightly loose stool; 2 = loose stool; 3 = slight diarrhea; 4 = severe diarrhea), and bloody stool (0 = none blood; 1 = little blood in stool; 2 = blood in stool; 3 = blood on anus; 4 = uncontrolled bleeding from the anus). DAI = combined score of weight loss, diarrhea, and bloody stool.

### Histological analysis of colon

For histological analysis, colons from indicated mice were fixed in 4% formalin and embedded in paraffin. Tissue sections 3-5 μm were stained with hematoxylin and eosin. The inflammation score was defined as follows: 0 = no infiltrate; 1 = occasional cell limited to submucosa; 2 = significant presence of inflammatory cells in submucosa, limited to focal areas; 3 = infiltrate present in submucosa, limited to focal areas; 4 = large amount of infiltrate in submucosa; 5 = transmural inflammation. The ulceration score was defined as follows: 0 = none; 1 = small, focal ulcers; 2 = frequent small ulcers; 3 = large areas lacking surface epithelium. The crypt damage score was defined as follows; 0 = none; 1 = some crypt damage, spaces between crypts; 2 = large spaces between crypts, some shortening of crypts; 3 = large areas without crypts, surrounded by normal crypts; 4 = no crypts.

### Quantitative reverse transcription PCR (qRT-PCR)

Total RNA was isolated from the indicated colonic epithelial cells and colon lamina propria with Trizol reagent RNAiso Plus (Takara, 9109) according to the manufacturer's protocol. Total RNA was reverse-transcribed to cDNA using the RevertAid First Strand cDNA Synthesis Kit (Thermo Fisher, USA, K1622). After reverse transcription, qPCR amplification was performed using Tip Green qPCR SuperMix (Transgen, China, AQ141-01) on a CFX96 Real-Time PCR System (Bio-Rad, USA). PCR programs consist of 95 °C for 10 min, 33 cycles of 95 °C for 15 s, and 1 min at 60 °C. The mRNA expression of target genes in each group of mice was determined by the 2^-∆Ct^ method relative to the expression of *Gapdh* or *β-actin.* Sequences of the primers used for PCR amplification are listed in Table S1.

### Immunofluorescence staining

Colon tissues from the indicated mice were paraffin-embedded and sectioned at a thickness of 7 μm. Tissue sections were incubated with dimethyl benzene for 15 min, followed by 85% and 75% ethanol treatments for 5 min. Deparaffinized tissue sections were subjected to antigen rederivation by EDTA buffer, and 3% BSA blockage before incubation with primary antibodies against Claudin1 (Servicebio, GB111032), Occludin (Servicebio, GB111401), Ly-6G (Servicebio, GB11229), and NK1.1 (Biolegend, PK136) overnight at 4 °C and washed with PBS for three times, followed by appropriate secondary antibody (Servcobio, GB21303, GG22404, GB22301) and DAPI (Serviceobio, G1012). Fluorescent Microscopy (Nikon, ECLIPSE C1) was used to identify the indicated protein expression. The TUNEL staining experiment was carried out using the Fluorescein (FITC) TUNEL Cell Apoptosis Detection Kit (Servicebio, G1501). The assay was conducted in accordance with the instructions.

### Isolation of colonic epithelial cells and lamina propria

The colonic epithelial cells were isolated by mechanical spin after 10 min digestion with 30 mM EDTA (Sigma-Aldrich, ED2SS-250G) in 37 °C water bath. Shed Epithelial cells from the colon were harvested and suspended with RPMI1640 medium before centrifuging at 1500 rpm for 5 min. The colonic epithelial cells were then processed for RNA isolation and protein extraction. The remaining colon segments were sliced and lysed with Trizol reagent (Takara, 9109) for additional RNA isolation as samples of colonic lamina propria.

### Western blotting analysis

The colonic epithelial cells were lysed with RIPA buffer (Solarbio, R0010) for protein extraction. Protein concentration was determined by the BCA assay kit (Beyotime, China, P0012). An equivalent quantity of protein samples was loaded on a 12% sodium dodecyl sulfate-polyacrylamide gel electrophoresis gel (Beyotime, P0012AC) and subsequently transferred to a polyvinylidene fluoride (PVDF) membrane (Merk Millipore, IPVH15150). Then the membrane was blocked with 5% skimmed milk, and it was incubated with primary antibodies against p38 (Cell Signaling Technology, 8690), Phospho-p38 (Cell Signaling Technology, 4511S), p65 (Cell Signaling Technology, 8242), Phospho-p-65 (Cell Signaling Technology, 3033), c-Fos (Biolegend, 641401), Phospho-c-Fos (Cell Signaling Technology, 5348) and β-actin (Cell Signaling Technology, 3700) overnight at 4 °C. After washing with Tris-buffered solution containing 0.05% Tween 20, the blots were incubated with secondary antibodies (Abcam, ab6271, ab6728) for two hours at room temperature. After five additional washes, the protein bands were visualized using an ECL reagent kit (Solarbio, PE0010) based on the manufacturer's instructions.

### Flow Cytometry

Mesenteric lymph nodes were collected and prepared for a single cell suspension. After Fc blockade by incubating anti-mouse CD16/32 (Biolegend, S17011E), cells were stained with fluorochrome-conjugated antibodies against CD3 (Biolegend, 17A2), CD4 (Biolegend, GK15), CD8a (Biolegend, 53-6.7), CD11b (Biolegend, M1/70), CD11c (Biolegend, N418), NK1.1 (Biolegend, PK136), F4/80 (Biolegend, BM8), Ly-6G (Biolegend, LA8), and Ly-6C (Biolegend, HK1.4). Live cells were identified with the LIVE/DEAD™ Fixable Near-IR Dead Cell Stain Kit (Thermo Fisher, USA). After washing with FACS buffer, the data was collected using an Attune NxT FCM flow cytometer (Invitrogen, USA) and analyzed with the FlowJo software (BD biosciences, USA).

### Statistical Analyses

GraphPad Prism software (version 5.0) was used for statistical analysis of data and graph generation. Data are expressed as mean and SEM, or as mean and SD. Statistical significance was evaluated using unpaired two-tailed Student's t-test with Welch's correction, one-way ANOVA with Dunnett's multiple comparisons test or unpaired two-tailed Student's test as indicated in the relative figure legends.

## Supplementary Material

Supplementary figures and table.

## Figures and Tables

**Figure 7:**
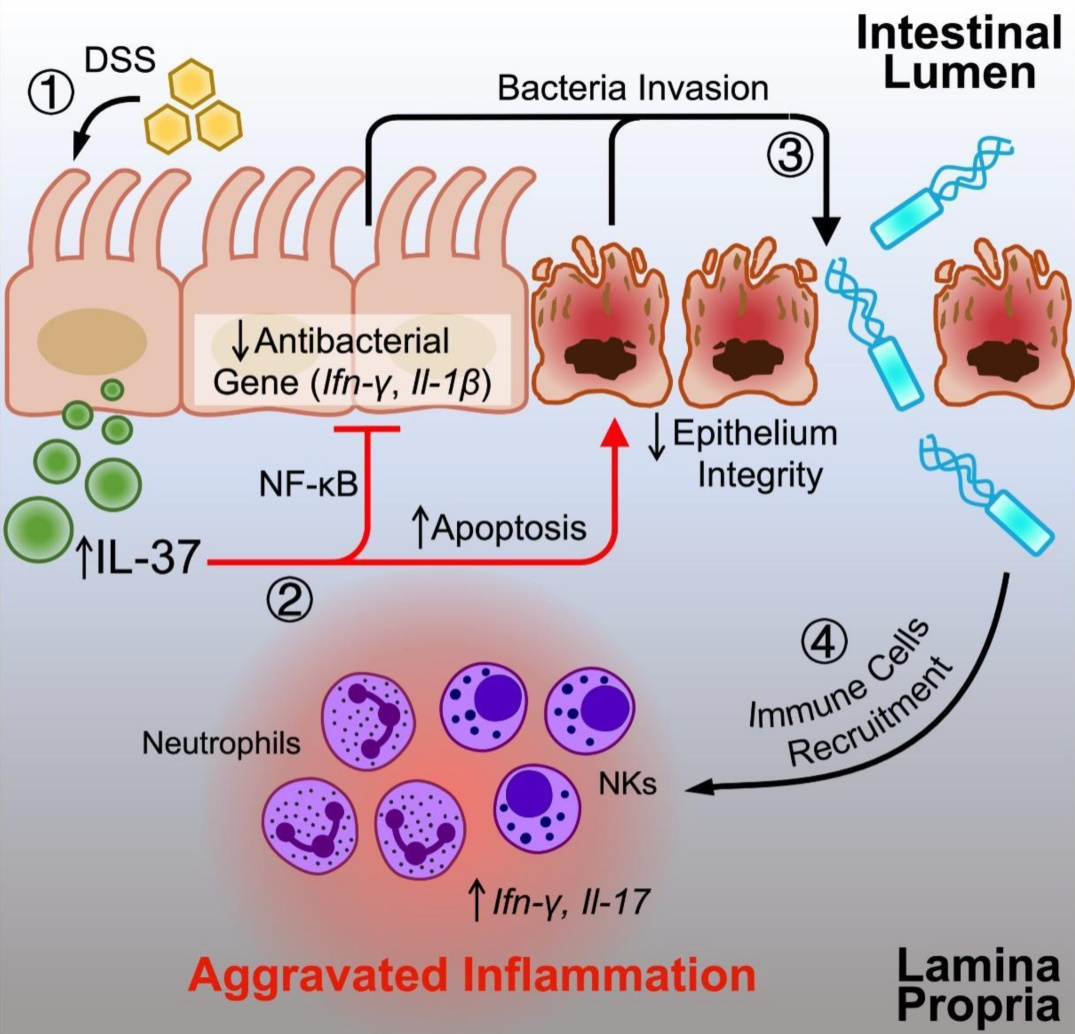
** The graph illustrates the potential influence of IL-37 on colitis.** DSS can induce IL-37 expression in intestinal epithelial cells under conventional housing conditions. Upregulated IL-37 increases epithelial cell apoptosis, inhibits NF-κB dependent antibacterial gene expression, and impairs epithelium integrity, resulting in an imbalance in intestinal homeostasis by promoting bacterial invasion. The disruption of the gut microbiota by IL-37 causes the migration of neutrophils and NK cells into the lamina propria, boosting the production of proinflammatory cytokines (IL-17 and IFN-γ) and accelerating intestinal inflammation.

## Abbreviations

IBD: Inflammatory bowel disease
CD: Crohn's disease
UC: ulcerative colitis
IL-37tg: Transgenic expressing human IL-37
DSS: dextran sulfate sodium
SPF: specific pathogen-free
IL-18Rα: IL-18 receptor α
IL-1R8: IL-1 receptor 8
IL: interleukin
WT: wild-type
IEC: Intestinal epithelial cells
TUNEL: terminal deoxynucleotidyl transferase dUTP nick-end labeling
IFN: Interferon
P-p65: phosphorylated-p65
MAPK: mitogen-activated protein kinase
NF-κB: nuclear factor κB
CLP: colon lamina propria
MLN: mesenteric lymph node
TNF^deltaARE^: a deletion in the tumor necrosis factor AU-rich (adenosin-uracil) elements (ARE)
E. coli: Escherichia coli
PCoA: principal coordinate analysis
Anosim: analysis of similarities
OTUs: operational taxonomic units
LEfSe: linear discriminant analysis effect size analysis measurements
DAI: disease activity index
