# Supplementary material for: Interleukin-37 exacerbates experimental colitis in an intestinal microbiome-dependent fashion
Source: Theranostics. 2022 Jul 4;12(11):5204–19. doi: 10.7150/thno.69616 (PMC9274733; doi:10.7150/thno.69616)
Supplement: Supplementary file 1 — Supplementary figures and table. [file thnov12p5204s1.pdf]

Supplementary Material

Interleukin-37 exacerbates experimental colitis in an intestinal microbiome-dependent fashion

Junxiao Cong[1,*], Dandan Wu[1,*], Hanying Dai[1,*], Yanmei Ma[1,2,*], Chenghui Liao[1,2], Lingyun Li[1], Liang Ye[1,#] and Zhong Huang[1,#]

[1]Department of Immunology, Biological Therapy Institute of Shenzhen University, Guangdong Provincial Key Laboratory of Regional Immunity and Diseases, International Cancer Center, Shenzhen University Health Science Center, Shenzhen, China.
[2]Shenzhen Futian Hospital for Rheumatic Diseases, Shenzhen, China.
[*]These authors contributed equally

[#]Corresponding author:
Zhong Huang, Email: zhuang809@126.com
Liang Ye, Email: liangyeszu@163.com

Supplementary Figures

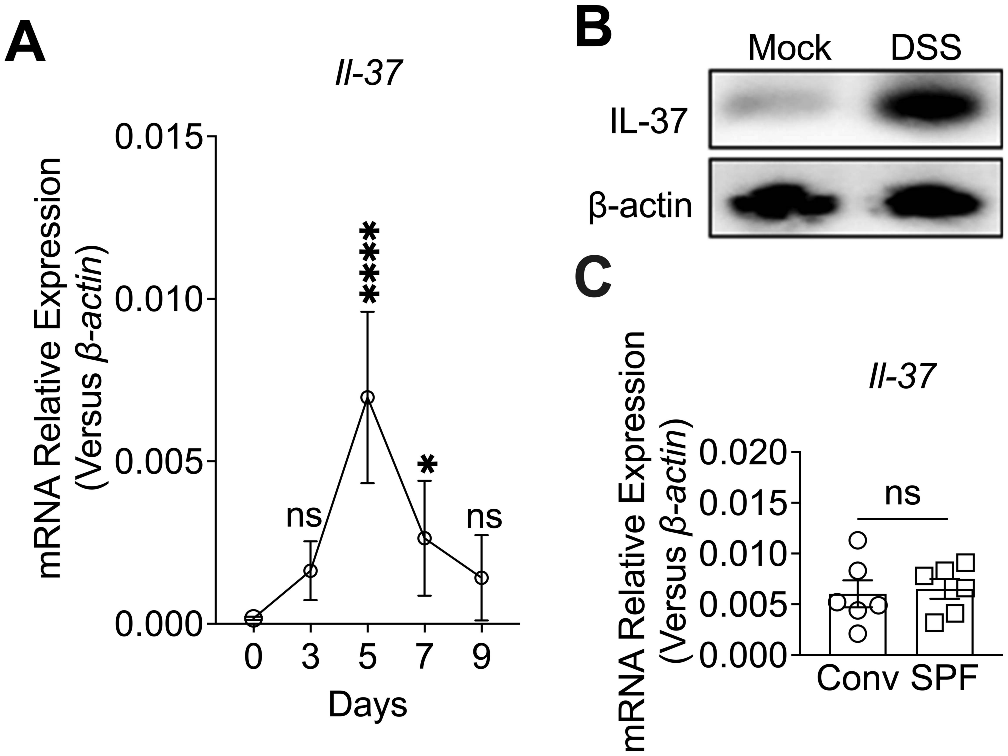

Figure S1. Constitutive expression of hIL-37 in mouse intestinal epithelial cells responsive to DSS induction. Conventionally housed IL-37tg mice were given 1.5% DSS in drinking water for nine days**. (A)** the levels of *Il-37* mRNA in colonic epithelial cells at different time points were detected by qRT-PCR. **(B)** On day five after administration of 1.5% DSS, western blot detection of IL-37 protein levels in the colonic epithelial cells of conventionally housed IL-37tg mice. **(C)** On day five after administration of 1.5% DSS, Il-37 mRNA expression in colonic epithelial cells of IL-37tg mice kept under conventional housing conditions and SPF conditions was determined by qRT-PCR. Data in (A) are shown as mean ± SD, and the data in (C) are shown as mean ± SEM. * $p < 0.05$, **** $p < 0.0001$ by one-way ANOVA with Dunnett's multiple comparisons test. ns = no significant difference by unpaired two-tailed Student's t-test with Welch's correction.

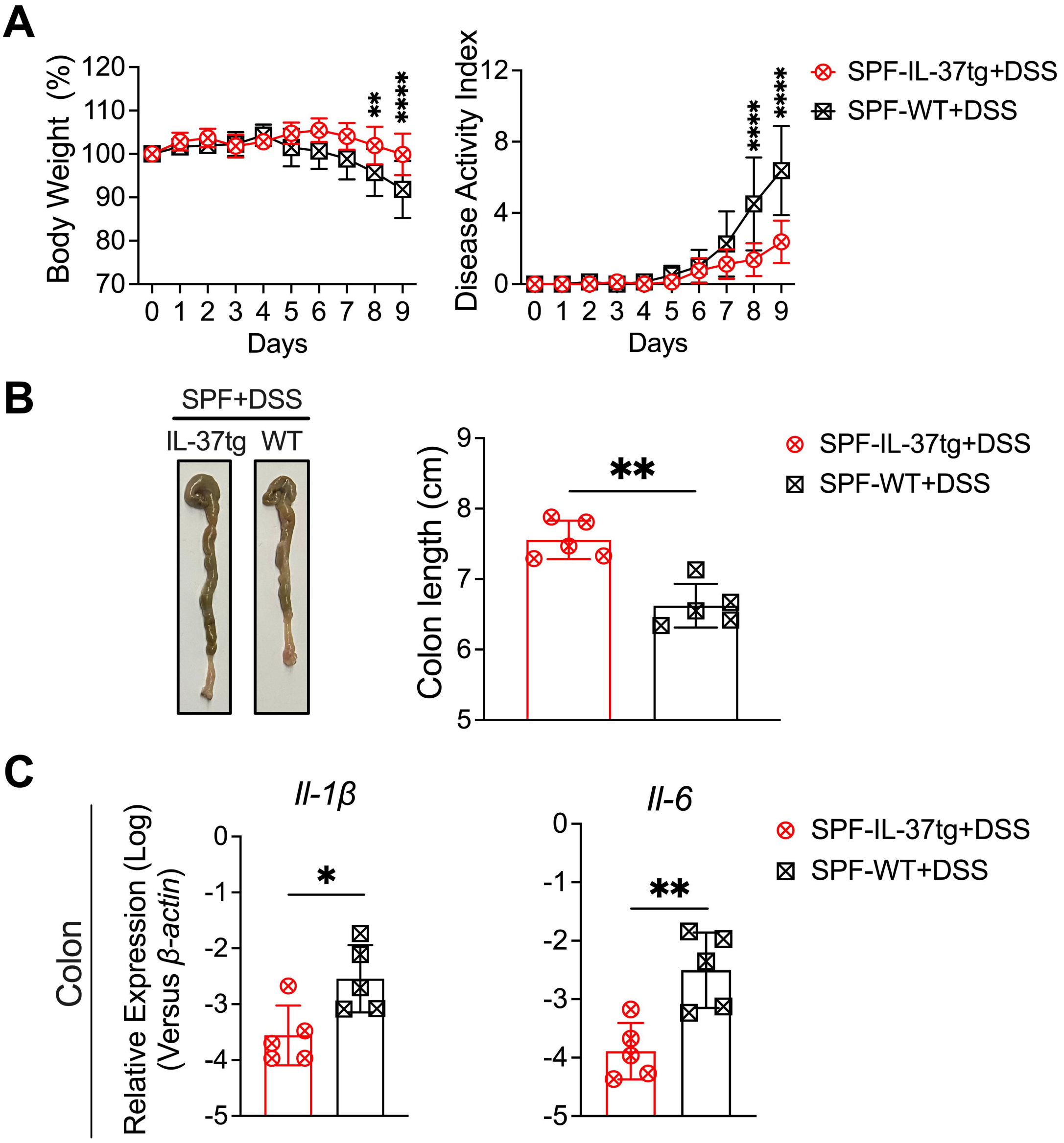

Figure S2. DSS-induced colitis is alleviated in SPF IL-37tg mice. SPF WT and IL-37tg mice were given drinking water with 1.5% DSS for eight days (n = 10 each group), followed by a 24-hour recovery period. **(A)** Between days 0 and 9, the body weight changes (left panel) and disease activity index changes (right panel) were evaluated daily. **(B)** Macroscopic images of representative mouse colons and assessed colon length. **(C)** *IL-1β* and *Il-6* mRNA levels in the colon are analyzed by qRT-PCR. The data in **(A)** are presented as mean ± SD, while the data in **(B-C)** are shown as mean ± SEM. * $p < 0.05$, ** $p < 0.01$, **** $p < 0.0001$, ns = no significant difference by two-way ANOVA with Sidak's multiple comparisons test and unpaired two-tailed Student's t-test with Welch's correction.

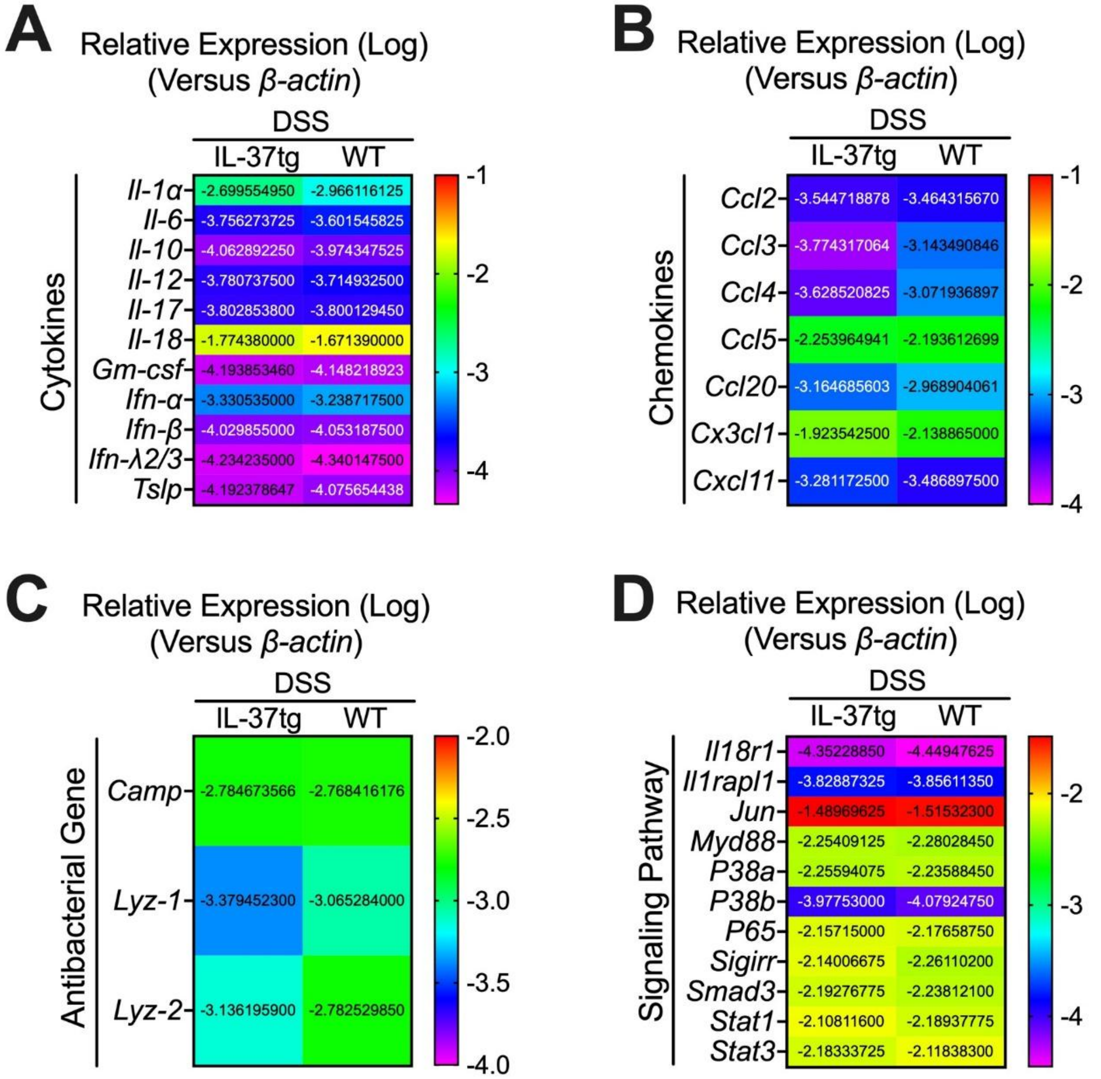

Figure S3. Heatmaps of gene expression variations in colonic epithelial cells from conventionally housed WT and IL-37tg colitis mice. On day nine, intestinal epithelial cells from conventionally housed WT and IL-37tg mice after 1.5% DSS supplementation in drinking water were analyzed by qRT-PCR to determine the gene expression of cytokines **(A)**, chemokines **(B)**, antibacterial factors **(C)**, and associated signaling pathways **(D)**. Red represents upregulated gene expression relative to the *β-actin*, while purple denotes downregulated gene expression compared to the *β-actin*. The values indicate the mean fold change, and the color intensity reflects the magnitude of the calculated fold change.

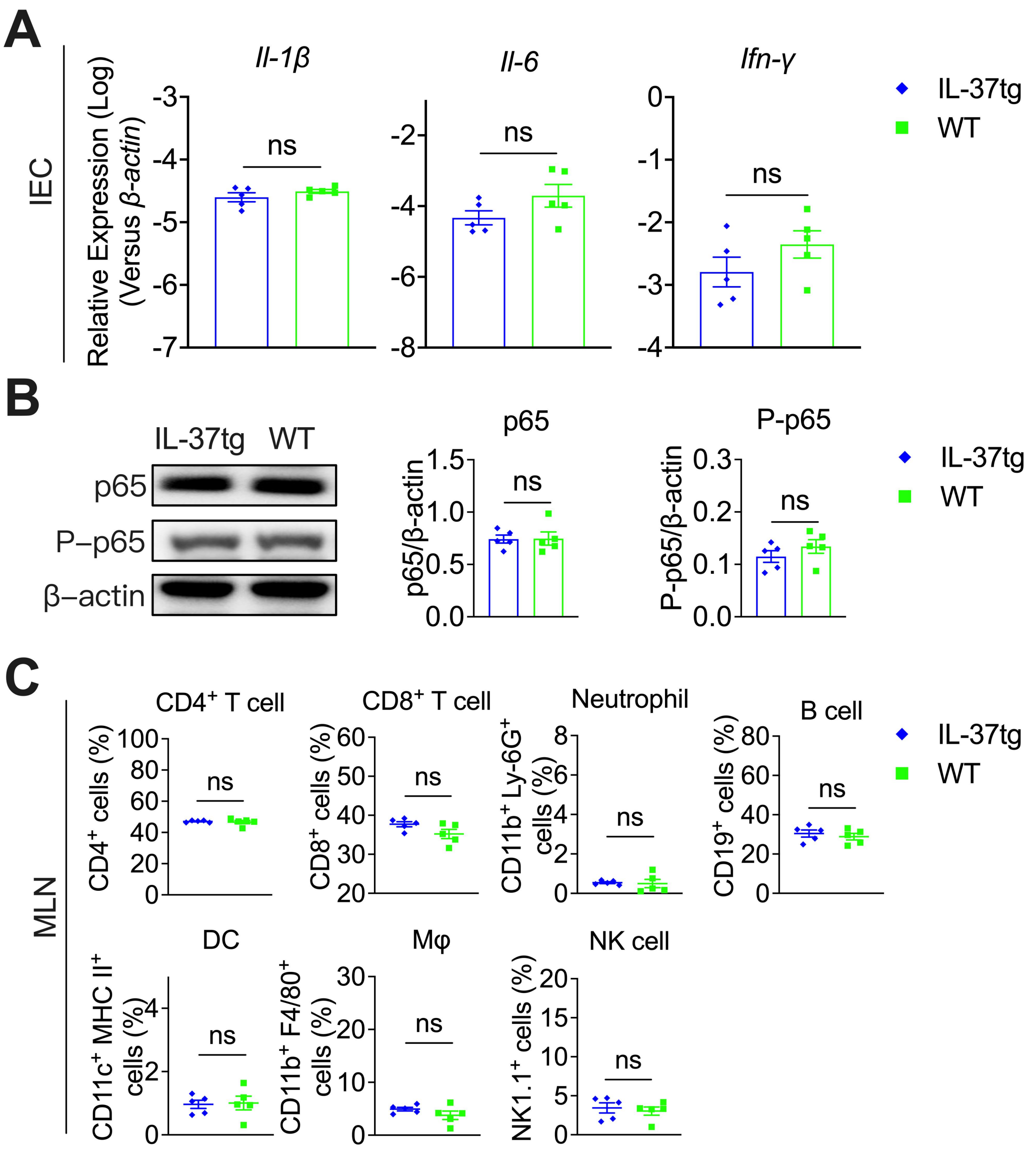

Figure S4. Overexpression of hIL-37 does not affect inflammatory cytokines in IECs or the immune cell profile in MLNs under static conditions. Intestinal epithelial cells (IECs) and mesenteric lymph nodes (MLNs) were isolated from conventionally housed IL-37tg (n = 5) and WT (n = 5) mice at static status. **(A)** qRT-PCR analysis of *Il-1β*, *Il-6*, and *Ifn-γ* mRNA expression in IECs. **(B)** NF-κB pathway proteins are detected by western blot. **(C)** The Immune cell composition in MLNs is determined by flow cytometry. Each symbol represents an individual animal. Data are shown as mean ± SEM. ns = no significant difference by unpaired two-tailed Student's t-test with Welch's correction.

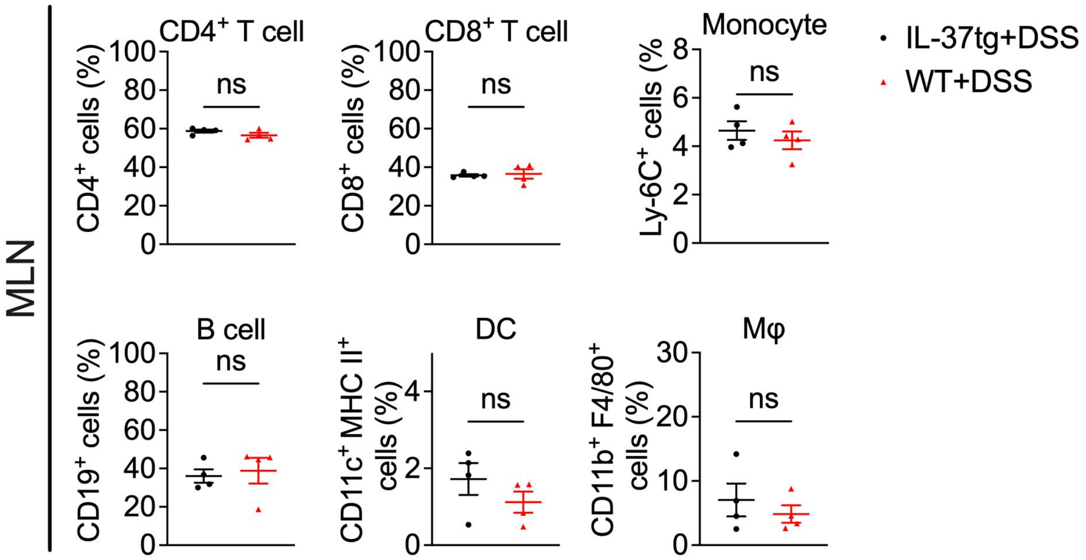

Figure S5. IL-37 does not alter the percentage of B cells, T cells, DCs, and macrophages in the MLN. Flow cytometric analysis of the proportion of $CD4^+$ T cells, $CD8^+$ T cells, $Ly6c^+$ monocytes, $CD19^+$ B cells, $CD11c^+MHCII^+$ DCs, and $CD11b^+F4/80^+$ macrophages in mesenteric lymph nodes (MLN) from 1.5% DSS-induced conventionally housed WT and IL-37tg mice at day nine. Data are shown as mean ± SEM. ns = no significant difference by unpaired two-tailed Student's t-test with Welch's correction.

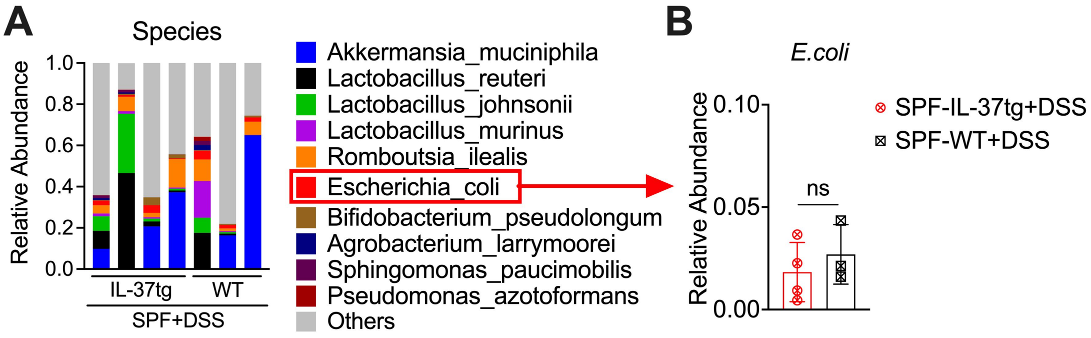

Figure S6. The relative abundance of *E. coli* in SPF IL-37tg colitis mice is unaffected. SPF WT and IL-37tg mice were given drinking water with 1.5% DSS for eight days, followed by a 24-h recovery period. Relative abundance of intestinal microbiota from DSS-treated SPF WT and IL-37tg mice on Species-level and *E. coli.* ns = no significant difference by unpaired two-tailed Student's t-test with Welch's correction.

Table S1. Primers used in this study

Genes	Forward primers (5'-3')	Reverse primers (5'-3')
Ifn-γ	CGGCACAGTCATTGAAAGCC	TGCATCCTTTTTCGCCTTGC
Il-1α	ACGGCTGAGTTTCAGTGAGACC	CACTCTGGTAGGTGTAAGGTGC
Il-1β	dCAGGATGAGGACATGAGCACC	CTCTGCAGACTCAAACTCCAC
Il-37(human)	TCAGCCTCTGCGGAGAAAGGAA	GCGTGCTGATTCCTTTTGGGCA
Il-6	TACCACTTCACAAGTCGGAGGC	CTGCAAGTGCATCATCGTTGTTC
Il-8	GGTGATATTCGAGACCATTTACTG	GCCAACAGTAGCCTTCACCCAT
Il-10	CGGGAAGACAATAACTGCACCC	CGGTTAGCAGTATGTTGTCCAGC
Il-17	CAGACTACCTCAACCGTTCCAC	TCCAGCTTTCCCTCCGCATTGA
Il-18	GACAGCCTGTGTTCGAGGATATG	TGTTCTTACAGGAGAGGGTAGAC
Gm-csf	AACCTCCTGGATGACATGCCTG	AAATTGCCCCGTAGACCCTGCT
Ifn-α	GGATGTGACCTTCCTCAGACTC	ACCTTCTCCTGCGGGAATCCAA
Ifn-β	GCCTTTGCCATCCAAGAGATGC	ACACTGTCTGCTGGTGGAGTTC
Ifn-λ2/3	CCAGTGGAAGCAAAGGATTGCC	TCAGGTCCTTCTCAAGCAGCCT
Inos	GAGACAGGGAAGTCTGAAGCAC	CCAGCAGTAGTTGCTCCTCTTC
Gapdh	CTCTCTGCTCCTCCCTGT	GCAACAATCTCCACTTTG
β-actin	AACAGTCCGCCTAGAAGCAC	CGTTGACATCCGTAAAGACC
Lyz1	GAGACCGAAGCACCGACTATG	CGGTTTTGACATTGTGTTCGC
Lyz2	ATGGAATGGCTGGCTACTATGG	ACCAGTATCGGCTATTGATCTGA
Ccl2	TAAAAACCTGGATCGGAACCAAA	GCATTAGCTTCAGATTTACGGGT
Ccl3	TGTACCATGACACTCTGCAAC	CAACGATGAATTGGCGTGGAA
Ccl4	TTCCTGCTGTTTCTCTTACACCT	CTGTCTGCCTCTTTTGGTCAG
Ccl5	GCTGCTTTGCCTACCTCTCC	TCGAGTGACAAACACGACTGC
Cxcl11	TGTAATTTACCCGAGTAACGGC	CACCTTTGTCGTTTATGAGCCTT
Ccl20	ACTGTTGCCTCTCGTACATACA	GAGGAGGTTCACAGCCCTTTT
Cx3cl1	CTGGCCGCGTTCTTCCATT	GCACATGATTTCGCATTTCGT
Tslp	ACGGATGGGGCTAACTTACAA	AGTCCTCGATTTGCTCGAACT
Mucin1	GGCATTCGGGCTCCTTTCTT	TGGAGTGGTAGTCGATGCTAAG
Camp	GCTGTGGCGGTCACTATCAC	TGTCTAGGGACTGCTGGTTGA
Il18r1	ACTTTTGCTGTGGAGACGTTAC	CCGGCTTTTCTCTATCAGTGAAT
Il1rapl1	ACTGATTGAATGCAGCGAACT	GGTCCATGCCATTTAATAACCGT
Sigirr	GTGACATGGCCCCTAATTTCC	ATGCCAGACCATCTTTCAGCC
Smad3	CACGCAGAACGTGAACACC	GGCAGTAGATAACGTGAGGGA
Stat3	CAATACCATTGACCTGCCGAT	GAGCGACTCAAACTGCCCT
Myd88	AGGACAAACGCCGGAACTTTT	GCCGATAGTCTGTCTGTTCTAGT
P65	AGGCTTCTGGGCCTTATGTG	TGCTTCTCTCGCCAGGAATAC
P38b	GCGGGATTCTACCGGCAAG	GAGCAGACTGAGCCGTAGG

P38a	TGACCCTTATGACCAGTCCTTT	GTCAGGCTCTTCCACTCATCTAT
Jun	CCTTCTACGACGATGCCCTC	GGTTCAAGGTCATGCTCTGTTT
Fos	CGGGTTTCAACGCCGACTA	TTGGCACTAGAGACGGACAGA
